# Influence of prehospital volume replacement on outcome in polytraumatized children

**DOI:** 10.1186/cc11809

**Published:** 2012-10-18

**Authors:** Bjoern Hussmann, Rolf Lefering, Max Daniel Kauther, Steffen Ruchholtz, Patrick Moldzio, Sven Lendemans

**Affiliations:** 1 Trauma Surgery Department, University Hospital Essen, Hufelandstraße 55, Essen 45122, Germany; 2 Institute for Research in Operative Medicine (IFOM), Faculty of Health, University of Witten/Herdecke, Cologne Merheim Medical Centre, Ostmerheimer Straße 200, Cologne 51109, Germany; 3 Department of Trauma, Hand and Reconstructive Surgery, University Hospital Giessen and Marburg, Baldingerstraße, Marburg 35043, Germany; 4 Clinic for Anesthesiology and Intensive Care, University Hospital Essen, Hufelandstraße 55, Essen 45122, Germany

## Abstract

**Introduction:**

Severe bleeding after trauma frequently results in poor outcomes in children. Prehospital fluid replacement therapy is regarded as an important primary treatment option. Our study aimed, through a retrospective analysis of matched pairs, to assess the influence of prehospital fluid replacement therapy on the post-traumatic course of severely injured children.

**Methods:**

The data for 67,782 patients from the TraumaRegister DGU^® ^of the German Trauma Society were analyzed. The following inclusion criteria were applied: injury severity score ≥16 points, primary admission, age 1 to 15 years old, systolic blood pressure ≥20 mmHg at the accident site and transfusion of at least one unit of packed red blood cells (pRBC) in the emergency trauma room prior to intensive care admission. As volume replacement therapy depends on age and body weight, especially in children, three subgroups were formed according to the mean value of the administered prehospital volume. The children were matched and enrolled into two groups according to the following criteria: intubation at the accident site (yes/no), Abbreviated Injury Scale (four body regions), accident year, systolic blood pressure and age group.

**Results:**

A total of 31 patients in each group met the inclusion criteria. An increase in volume replacement was associated with an elevated need for a transfusion (≥10 pRBC: low volume, 9.7%; high volume, 25.8%; *P *= 0.18) and a reduction in the ability to coagulate (prothrombin time ratio: low volume, 58.7%; high volume, 55.6%; *P *= 0.23; prothrombin time: low volume, 42.2 seconds; high volume, 50.1 seconds; *P *= 0.38). With increasing volume, the mortality (low volume, 19.4%; high volume, 25.8%; *P *= 0.75) and multiple organ failure rates (group 1, 36.7%; group 2, 41.4%; *P *= 0.79) increased. With increased volume, the rescue time also increased (low volume, 62 minutes; high volume, 71.5 minutes; *P *= 0.21).

**Conclusion:**

For the first time, a tendency was shown that excessive prehospital fluid replacement in children leads to a worse clinical course with higher mortality and that excessive fluid replacement has a negative influence on the ability to coagulate.

## Introduction

Accidents are still a major cause of death in children [1]. Apart from severe traumatic brain injury, uncontrolled bleeding and the corresponding hemorrhagic shock play significant roles [2-4]. Blunt trauma is the most common form of severe trauma in Europe (96% in Germany, according to the TraumaRegister DGU^® ^2011 annual report). Blunt trauma that causes bleeding into the large (thoracic and/or abdominal) body cavities is especially difficult to assess diagnostically. Furthermore, these injuries have been correlated with increased mortality rates [5-8].

At first glance, a reasonable course of action appears to be the replacement of lost blood with fluids as quickly as possible; that is, at the accident site [9]. However, no studies have confirmed that the immediate administration of fluids is beneficial to trauma patients with internal bleeding. Unlike assessments of blunt trauma, the influence of prehospital fluid replacement on penetrating injuries has been more thoroughly investigated in adults. Studies involving patients who suffered penetrating injuries have shown that excessive replacement volumes (>2,000 ml), which also result in longer times from the accident to arrival at the hospital, are correlated with increased mortality rates after trauma in most cases [10,11].

A recent study has also shown in cases of blunt trauma that non-indicated enhanced volume replacement therapy is associated with increased mortality [12]. Furthermore, Rourke and colleagues demonstrated that increased prehospital volume administration is associated with reduced fibrinogen blood levels that entail a negative outcome for the patient [13]. On the basis of multivariate regression analysis, Haut and colleagues have shown retrospectively that prehospital volume replacement therapy represents an independent risk factor [14].

However, all of the above-mentioned studies and recommendations were established on the basis of an adult patient cohort. Concerning severely injured, bleeding children, there are currently no clear recommendations or studies with a high evidence level in the literature. The current S3 guidelines of the German Trauma Society for the most severely injured patients also do not comment on volume replacement therapy in children at the accident site [15]. Some studies have recommended rather aggressive volume replacement therapy after trauma. Here, on the one hand, volume replacement therapy is meant to improve the perfusion pressure, and, on the other, organ perfusion is meant to be sustained [16].

In the current literature, complications have also been reported after enhanced volume replacement therapy in children. One case report described the formation of abdominal compartment syndrome after extensive volume replacement therapy [17].

Several questions arise after an examination of the current literature, including the following: does the quantity of volume that is replaced have consequences for hemorrhagic shock in the post-traumatic course, including multiple organ failure, sepsis, outcomes and mortality in the most severely injured, bleeding children? We addressed this question in a cohort of children that was selected from the TraumaRegister DGU^® ^of the German Trauma Society and that had suffered severe injuries (Abbreviated Injury Scale (AIS) >3) which resulted in hemorrhaging.

## Materials and methods

The TraumaRegister DGU^® ^of the German Trauma Society (Deutsche Gesellschaft für Unfallchirurgie (DGU)) was initiated in 1993 for the purpose of comparative quality audits. The register contains prospectively collected data from 367 collaborating European trauma centers, mostly in Germany. The data were entered by hand from patient records until 2001, when data input was automated for central submission via web-based data entry software (since 2002). Approximately 100 data points per patient have been collected, including the coding of each injury according to the AIS (revised version of 1998). The data are submitted to a central database hosted by the Academy for Trauma Surgery (Akademie der Unfalchirurgie GmbH). Anonymity of the data is guaranteed for both the patient and the participating hospital.

Only patients from Germany and Austria were included in this study to minimize variations due to different rescue systems. All of the patients were attended to by a physician prior to hospital admission. Records that were collected between 1993 and 2010 (67,782 patients) were considered for this study. The trauma registry is a voluntary register, is approved by the review board of the German Society for Trauma Surgery and is in compliance with institutional requirements. As the trauma registry of the DGU is an anonymous register, the Institution Review Board waived the need for patient consent. The data for the trauma registry of the DGU have received the full approval of the ethics committee of the University of Witten/Herdecke, Cologne, Germany. The patients were selected for this study according to the following criteria: primary admission to the hospital (no transfers); Injury Severity Score (ISS) ≥16; age 1 to 15 years; infusion of at least one unit of packed red blood cells (pRBC) in the emergency trauma room prior to ICU admission; systolic blood pressure at the accident site ≥20 mmHg; and data available for prehospital administered fluid volume, on-scene time, hemoglobin concentration on hospital admission and blood pressure at the accident site and upon hospital admission.

Initially, we assigned the patients to one of three age groups based on the physiological development of the children and on associated therapy adjustments (for example, with regard to prehospital volume administration): Group 1, 1 to 4 years old (small child); Group 2, 5 to 10 years old (school child); and Group 3, 11 to 15 years old (adolescence).

**Figure 1:**
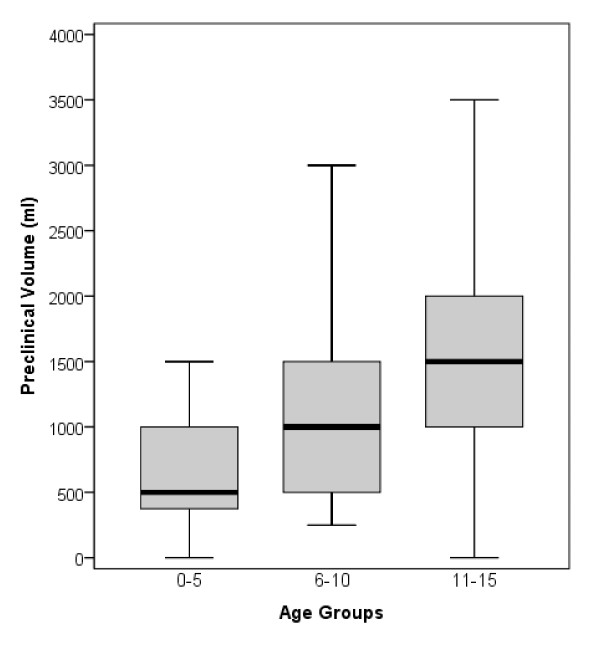
**Box plot of preclinical volume in the three age groups**. Sample sizes were 36, 25 and 115, respectively.

According to the prehospital administered fluid volume (crystalloids plus colloids), the patients were divided into a low-volume group and a high-volume group on the basis of the amount of the prehospital administered volume in all children in the age groups: Group 1, low volume 0 to 500 ml, high volume >500 ml (mean value of all children in the trauma registry 500 ml); Group 2, low volume 0 to 1,000 ml, high volume >1,000 ml (mean value of all children in the trauma registry 1,000 ml); and Group 3, low volume 0 to 1,500 ml, high volume >1,500 ml (mean value of all children in the trauma registry 1,500 ml) (Figure 1).

To evaluate the effects of prehospital volume administration, patients receiving high-volume and low-volume fluid replacement were matched according to the following criteria. The first criterion was the pattern of injury for the following four body regions: head, thorax, abdomen and extremities (including the pelvis), for which the matching criterion was AIS severity ≥3 or <3 points. As a second criterion, to take into account changes in treatment that might have occurred over the years, the patients were divided into four groups according to the year of accident: 1993 to 2001; 2002 to 2004; 2005 to 2007; and 2008 to 2010. A third criterion, systolic blood pressure at the accident site, was subdivided into three groups: 20 to 89 mmHg; 90 to 99 mmHg; and ≥100 mmHg. Age was matched according to the three subgroups: 1 to 4 years old; 5 to 10 years old; and 11 to 15 years old. The final criterion was preclinical intubation (yes/no).

Sepsis was defined according to the criteria of Bone, which resemble the American College of Chest Physicians/Society of Critical Care Medicine consensus conference definition [18]. Single organ failure was defined as a score ≥3 points on the Sequential Organ Failure Assessment [19]. Multiple organ failure was defined as simultaneous organ failure in at least two organs. Prehospital parameters, length of hospital stay and coagulation parameters were examined separately in each group. For coagulation, the prothrombin ratio is a parameter that is commonly used in Germany and that corresponds to the International Normalized Ratio. To evaluate injury severity within the groups, prognostic estimation was performed by means of the Revised Injury Severity Classification (RISC) [20] and the Trauma and Injury Severity Score (TRISS). Prognoses were then compared with the observed mortality rates in the corresponding groups. To determine the probability of mass transfusion, the group-specific Trauma Associated Severe Hemorrhage score was calculated [21].

### Statistical analysis

The data were analyzed with the Statistical Package for the Social Sciences (version 17; SPSS Inc., Chicago, IL, USA). Incidences are represented as numbers of cases and percentages, and continuous values are presented as means and standard deviations (SDs). Differences between the two groups with low and high prehospital volumes were evaluated using the McNemar test in cases of dichotomous variables, the Wilcoxon signed-rank test for ordered categories, and the paired *t *test for continuous variables. In cases of obvious deviation from normality, continuous variables were also tested with the Wilcoxon test. *P *<0.05 was considered statistically significant.

## Results

From the TraumaRegister DGU^®^, 176 children met the inclusion criteria. Among these, 31 severely injured children in the high-volume group were matched with 31 children in the low-volume group. The mean patient age in the overall group was 11.3 years (SD 4.5). The ISS was not significantly different between the groups (low volume, 34.7 (SD 12.2); high volume, 37.3 (SD 14.4); *P *= 0.34). As expected, most injuries were blunt trauma injuries (96.8%). As previously expected, the matching showed that the subdivision of injury severity in the corresponding body regions had identical distributions between the groups (Table 1). The similarity of the general characteristics after matching the patients in this study substantiates that the groups receiving low-volume or high-volume replacement therapy were similar and comparable.

**Table 1:** Demographic and clinical data for bleeding, severely injured children treated with fluid replacement therapy

Patient characteristic	Low-volume group	High-volume group	Group mean (all patients)	*P *value
Patients (*n*)	31	31	62	
Age (years)	11.1 (4.5)	11.4 (4.6)	11.3 (4.5)	0.34
Male (%)	74.2	51.6	62.9	0.09
Glasgow coma scale	8.0 (4.4)	7.3 (4.6)	7.7 (4.5)	0.61
Injury severity score	34.7 (12.2)	37.3 (14.4)	36.0 (13.3)	0.34
Blunt trauma (%)	100	93.5	96.8	0.50
AIS head	3.4 (1.8)	3.4 (1.8)	3.4 (1.8)	0.56
AIS thorax	2.1 (1.8)	2.1 (1.9)	2.1 (1.8)	0.96
AIS abdomen	1.8 (1.8)	1.6 (1.9)	1.7 (1.9)	0.33
AIS extremities including pelvis	2.1 (1.5)	2.2 (1.5)	2.2 (1.5)	0.88

Demographic and clinical data on bleeding, severely injured children treated prior to hospitalization with low-volume or high-volume fluid replacement therapy (31 patients per group). Data shown as the mean (standard deviation) or percentage of the group. AIS, Abbreviated Injury Scale.

### Prehospital and emergency department treatment

As expected based on matching criteria, less fluid was infused in the low-volume group (on average, 863 ml) than in the high-volume group (on average, 2,137 ml) prior to arrival at the hospital. The percentage of children with penetrating injuries was larger in the high-volume group (low volume, 0%; high volume, 6.5%; *P *= 0.50). At the accident site, systolic blood pressure was lower in the low-volume group (low volume, 108 mmHg (SD 16); high volume, 112 mmHg (SD 19); *P *= 0.44). Upon arrival at the hospital, there was no significant difference in systolic blood pressure between the two groups (Table 2). Additionally, the heart rate and breathing rate at the accident site and upon arrival at the hospital did not show any significant differences (Table 2). Taken together, the clinical parameters for circulation were similar in both patient groups.

**Table 2:** Data for prehospital and emergency department treatment in children on fluid administration

Patient characteristic	Low-volume group	High-volume group	Group mean (all patients)	*P *value
Number of cases	31	31	62	
Fluid volume replaced prehospital (ml)	863 (433)	2,137 (873)	1,500 (938)	<0.001
Fluid volume replaced until end in trauma room (ml)	2,632 (2099)	3,334 (2649)	2,977 (2390)	0.18
Transport - emergency rescue helicopter (%)	67.7	61.3	64.5	0.80
Total prehospital time (minutes)	62.0 (17.7)	71.5 (32.7)	66.8 (26.5)	0.21
Blood pressure at accident site (mmHg)	108 (16)	112 (21)	110 (19)	0.44
Blood pressure at admission to hospital (mmHg)	108 (16)	108 (22)	108 (19)	0.91
Respiratory rate at the accident site	14.4 (7.3)	14.9 (6)	14.7 (6.5)	0.71
Heart rate at accident site (seconds)	102 (26)	104 (22)	103 (24)	0.69
Heart rate at admission to hospital (seconds)	106 (22)	98 (32)	102 (27)	0.45
Temperature upon arrival in the trauma room (°C)	35.5 (1.1)	36.0 (1.1)	35.7 (1.1)	0.88
Hemoglobin at admission to hospital (mg/dl)	8.0 (4.4)	7.2 (4.7)	7.7 (4.5)	0.10
Prothrombin ratio in hospital (%)	58.7 (19.4)	55.6 (21.9)	57.3 (21.9)	0.23
Prothrombin time in hospital (seconds)	42.2 (16.3)	50.1(34.4)	45.9 (26.3)	0.38
Base excess in hospital	-3.2 (4.2)	-5.5 (6.7)	-4.2 (5.5)	0.40
Units of pRBC in hospital	5.6 (5.7)	6.9 (7.1)	6.3 (6.5)	0.43
Massive transfusions ≥10 units pRBC until ICU admission (%)	9.7	25.8	17.7	0.18
Units of fresh-frozen plasma in hospital	3.0 (4.5)	2.4 (4.5)	2.8 (4.5)	0.36
Transfusion of thrombocytes in the trauma room (%)	12.9	16.1	14.5	1.00
Prehospital use of catecholamines (%)	9.7	13.3	11.5	1,00
Prehospital intubation (%)	96.8	96.8	96.8	1.00
Prehospital chest tube (%)	3.2	3.3	3.3	1.00
Prehospital cardiopulmonary resuscitation (%)	3.2	3.2	3.2	1.00
Prehospital sedation (%)	100	100	100	1.00

Data from children on fluid administration at the accident site, in the emergency department and during initial surgical treatment. Data shown as the mean (standard deviation) or percentage of the group. pRBC, packed red blood cells.

Hemoglobin concentrations and the base excess and coagulation values were measured during treatment in the emergency department. The patients who received high fluid volumes showed lower blood values; the hemoglobin concentration was lower in the high-volume group (low volume, 8 mg/dl (SD 4.4); high volume, 7.2 mg/dl (SD 4.7); *P *= 0.10) (Table 2). Similar results were observed for the prothrombin ratio (low volume, 58.7% (SD 19.4); high volume, 55.6% (SD 21.9); *P *= 0.23) and prothrombin time (low volume, 42.2 seconds (SD 16.3); high volume, 50.1 seconds (SD 34.4); *P *= 0.38). Patients in the high-volume group received more units of pRBC (low volume, 5.6 units (SD 5.7); high volume, 6.9 units (SD 7.1); *P *= 0.43). A higher percentage of patients in the high-volume group received more than 10 units of pRBC (low volume, 9.7%; high volume, 25.8%; *P *= 0.18) and thrombocyte concentrates (low volume, 12.9%; high volume, 16.1%; *P *= 1.00) (Table 2).

As shown in Table 2, patients receiving high-volume fluid replacement required more catecholamine supplementation. There were no significant differences between the groups regarding the percentages of patients who required cardiopulmonary resuscitation, sedation or insertion of a chest tube at the accident site (Table 2). The prehospital rescue time was longer in the high-volume group (low volume, 62 minutes; high volume, 71.5 minutes; *P *= 0.21).

### Clinical course and outcome

The days spent in the ICU were similar in both groups (Table 3). There was a significant difference concerning the total length of the hospital stay (low volume, 33.6 days; high volume, 23.3 days; *P *= 0.025). The number of ventilator-free days in the first 30 days (low volume, 18.1days; high volume, 14.6 days; *P *= 0.35) were different, but not significantly so (Table 2). The occurrences of sepsis, organ failure and multiple organ failure did not differ significantly between the two groups (Table 3).

**Table 3:** Clinical course and outcomes of bleeding in severely injured children treated with fluid replacement therapy

Patient characteristic	Low-volume group	High-volume group	Group mean (all patients)	*P *value
Stay in ICU (%)	96.8	96.8	96.8	1.00
Days in the ICU	13.9 (15.2)	14.0 (15.2)	13.9 (15.1)	0.88
Ventilator-free days (per first 30 ICU days)	18.1 (10.1)	14.6 (11.9)	16.4 (11.3)	0.35
Organ failure (%)	56.7	55.2	55.9	1.00
Multiple organ failure (%)	36.7	41.4	39.0	0.79
Sepsis (%)	14.3	11.5	13.0	1.00
RISC prognosis (%)	22.8	29.4	26.1	0.25
TRISS prognosis (%)	28.2	33.3	31.2	0.17
TASH score (point value)	18.7	32.2	24.2	0.025
Died in hospital (%)	19.4	25.8	22.6	0.75
Died within the first 6 hours (%)	6.5	9.7	8.1	1.00
Died within the first 24 hours (%)	9.7	12.9	11.3	1.00
Days of hospitalization	33.6 (28.9)	23.3 (20.2)	28.4 (25.3)	0.025

Clinical course and outcomes of bleeding, severely injured children receiving low-volume or high-volume prehospital replacement therapy after trauma and bleeding. Data shown as the mean (standard deviation) or percentage of the group. RISC, Revised Injury Severity Classification; TASH, Trauma Associated Severe Hemorrhage; TRISS, Trauma and Injury Severity Score.

TRISS prognosis showed no significant difference between the low-volume and high-volume groups. The same was true for RISC prognosis; however, there was a higher probability of death for patients in the high-volume group (29.4% compared with 22.8% in the low-volume group, *P *= 0.25; Table 3). RISC prognosis is based on values that are collected in the hospital, including the prothrombin ratio, hemoglobin concentration and administered pRBC. However, these values are directly influenced by the administered prehospital volume. The percentage of patients who died was higher in the high-volume group (25.8%) than in the low-volume group (19.4%, *P *= 0.75), but this difference was not significant (Table 3).

Trauma Associated Severe Hemorrhage scores showed a significantly higher probability for mass transfusion in the high-volume group (low volume, 18.7 points; high volume, 32.2 points; *P *= 0.025).

## Discussion

The present study focused on severely injured children who had hemorrhages. As such a patient cohort is rare even in a large registry, such as the TraumaRegister DGU^®^, it was not possible to show significant differences. Nevertheless, our study showed possible connections among increased volume replacement, impairment of the coagulation system and hemoglobin concentration upon arrival at the hospital. This finding was also illustrated by the number of units of transfused pRBC necessary for children receiving either low or high volumes of prehospital replacement therapy, and was especially apparent and clinically relevant in patients who received more than 10 units of pRBC. This relation has also been shown in a recent study with adults [12]. Again, however, there were no significant differences shown in our study. Unfortunately, these results could not be discussed due to a lack of current literature referring to severely injured children.

The decision for enhanced volume replacement therapy, which is initially administered at the accident site, must be made on a case-by-case basis. A comprehensive standard protocol cannot be established for these situations. However, the prolonged rescue time in the high-volume group did perhaps result in delayed therapy and, therefore, in hemorrhaging, especially in cases of blunt trauma. This therapy can only be administered in a hospital with the possibility of surgical therapy and optimal coagulation therapy. In this respect, previous studies with adults have shown that limiting prehospital therapy to the stabilization of the cardiovascular and pulmonary systems and prioritizing rapid transport to a level 1 trauma center are advantageous [12,22,23].

The reason why the children in the high-volume group received much larger volumes than patients in the low-volume group remains unknown. The classification was established so that the initial hemodynamic conditions would be approximately identical, as was the injury severity per body region. One possible bias arising from the distribution of dissimilar injury severities was thus minimized. In addition, there was no significant difference in the mean ISS between the groups. As mentioned earlier, the individual decision of what volume should be administered rests with the attending personnel. With regard to hemodynamic stability, only those patients who had systolic blood pressure ≥20 mmHg at the accident site were included in this study. Because it is assumed that patients with systolic blood pressure <20 mmHg receive larger volumes of solution at the accident site, these patients were not investigated due to the lack of a matching control group. It remains remarkable that there were more children with penetrating injuries in the high-volume group. Based on the current literature on penetrating trauma (for example, [11]), this way of proceeding cannot be explained. In the current literature, all authors agree that, for the purpose of permissive hypotension, short rescue times and low volumes seem to be reasonable in cases of penetrating injuries [10,11,22]. Why these children received greater volumes remains speculative, and this question cannot be resolved in individual cases by a retrospective registry study with anonymized patients. Individual assessments of the ambulance service personnel could play a role in the replacement volumes. This possibility, however, remains speculative and cannot currently be assessed due to a lack of relevant studies; therefore, individual assessment should be considered in future studies. The determining factors were possibly the education and experience of the medical personnel. This assumption is supported by a review from Oestern concerning medical assistance for severely injured patients in emergency trauma departments [24].

The results show that patients nonetheless receive cardiopulmonary resuscitation at the accident site. One must note that the systolic blood pressure used for matching referred to the blood pressure that was initially measured at the accident site. As mentioned before, diagnosis and subsequent therapy are subject to continuous changes and the possible worsening of the patient's condition. However, no conclusions about individual decisions can be drawn in a retrospective statistical analysis.

One remarkable finding of this study is that a higher replacement volume was related to a higher mortality rate. One can assume, as indicated by the data in this study, that increased prehospital replacement volumes and corresponding prolonged rescue times are responsible for the higher mortality rate after trauma because of, for example, impairment of the coagulation system. The possible treatments for these impaired conditions seem to be limited to the initial phase of treatment after trauma. Again, it must be emphasized that the results did not show any significant difference. No significant differences arose regarding sepsis, organ failure or multiple organ failure rates because of the increased mortality rate in the high-volume group. Similar results were obtained for the total days spent in the ICU and ventilator-free days over the first 30 days. Reduced volume replacement with the least possible additional impairment in coagulation, dilution of oxygen carriers and rapid transport to a level 1 trauma center for definitive surgical and intensive medical therapy appear to be the best courses of action [12]. In a recent study conducted in the USA, Haut and colleagues drew similar conclusions. Haut and colleagues postulated that the routine use of prehospital volume replacement must be avoided because it is associated with increased mortality. As a limitation, it should be noted that the emergency system on which Haut and colleagues' study was based is different from that in our study. While the ISS was split into four groups, no organ-specific matching (for example, using the AIS) was performed. Additionally, the average time from accident to hospital and the volume of administered solutions were not reported in this study [14].

The RISC score confirmed the influence of fluid volume on mortality because this score was directly influenced by the administered prehospital volume; that is, by the prothrombin ratio, hemoglobin concentration and transfusion of pRBC. Additionally, the Trauma Associated Severe Hemorrhage score, which is calculated on the basis of values that are directly influenced by the prehospital administered volume, such as the prothrombin ratio, reflected a significant greater probability of mass transfusion in the high-volume group. As already mentioned above, the severity of injuries in the individual body regions or the total ISS cannot alone be held responsible for higher mortality.

## Limitations

TRISS calculations could only be performed in 46% of the participating trauma centers, whereas the RISC methodology was available for 88% of the cases. The data might thus have been biased, as TRISS could not be calculated for the majority of trauma cases. However, this information also indicates that RISC is much easier to calculate than TRISS, possibly because RISC does not determine the prehospital respiratory rate. The respiratory rate is documented by the physicians at the accident sites in only 60% of cases.

Regarding the coagulation analysis, only the prothrombin ratio and prothrombin time are documented in the TraumaRegister DGU^® ^of the German Trauma Society and are available for analysis. Other laboratory values that might be of interest for coagulation (for example, fibrinogen and protein C) are not documented in the TraumaRegister DGU^®^.

All of the patients were treated by physicians at the accident sites. However, it remains unclear which specialties (for example, trauma surgeon, anesthetist) the physicians at the accident sites represented. For example, in Scandinavian countries, only anesthetists are allowed to work as physicians at accident sites. In German-speaking countries, any physician (for example, surgeon, anesthetist) with additional certification in emergency medicine is authorized to work as an emergency physician at accident sites. This certification is not comparable with the emergency physician certifications in most European countries or in the USA. In these countries, emergency physicians represents separate specialty. Furthermore, the individual decisions of the emergency physician remain unclear due to the lack of data in the trauma register. For the same reason, whether the on-site personnel had chosen appropriate cuff sizes for the appropriate measurement of systolic blood pressures in individual patients remains unclear. However, one must assume that the on-site personnel were taking appropriate measures (for example, selecting appropriate cuff sizes) in most of the cases. This has been shown in quality reviews conducted by the TraumaRegister DGU^®^.

Matched-pairs analysis is dependent on the quality of the matching criteria. When the patients are matched, not all of the patients in the trauma register are included because patients without a partner are not included. The advantage of comparing the patients included in the matched-pairs analysis, however, is that small differences can be demonstrated.

Finally, we only conducted a retrospective analysis; therefore, only associations (not causalities) could be ascribed to the given data. In the future, a prospective, randomized study will be indispensable in clarifying the advantages or disadvantages of a particular volume therapy at the accident site for the most severely injured, bleeding children.

## Conclusion

The present study has shown for the first time that, in cases of the most severely injured children in hemorrhagic shock, non-indicated aggressive volume replacement therapy has a negative influence on the clinical course and can perhaps result in higher mortality. Furthermore, non-indicated enhanced volume replacement therapy causes early traumatic coagulopathy. Despite the high number of patients in the TraumaRegister DGU^® ^(67,782 patients), the number of cases for the most severely injured children in hemorrhagic shock was so small it was not possible to demonstrate significant results. As there most probably will not be a larger cohort of cases, at least not in the German-speaking countries or in Europe, statements must always be made cautiously.

## Key messages

• Prehospital volume replacement in the most severely injured children is associated with a number of risks and should be critically weighed, except in cases where there are absolute indicators, such as severe traumatic brain injury.

• When applied uncritically, prehospital volume replacement in children after trauma can have a negative effect on the clinical course (for example, higher rate of multiple organ failure and mortality).

• Owing to the extended rescue time, an uncritically applied prehospital volume replacement in the most severely injured children leads to a delayed in-hospital patient care.

• Owing to a non-indicated prehospital volume replacement in children after trauma, the coagulation system is negatively affected and therefore the starting conditions in hospital deteriorate.

## Abbreviations

AIS: Abbreviated Injury Scale; DGU: Deutsche Gesellschaft für Unfallchirurgie (German Association for Trauma Surgery); ISS: Injury Severity Score; pRBC: packed red blood cells; RISC: Revised Injury Severity Classification; SD: standard deviation; TRISS: Trauma and Injury Severity Score.

## Competing interests

The authors declare that they have no competing interests.

## Authors' contributions

BH, PM, MDK and SL conceived the study, designed the trial, and obtained research funding. SL, SR and RL supervised the conduct of the trial and data collection. RL, BH and SL provided statistical advice on the study design and analyzed the data. BH drafted the manuscript, and all authors contributed substantially to its revision. BH takes responsibility for the paper as a whole. All authors read and approved the final manuscript for publication.
